# Supplementary figures and images for: Effect of high-flow nasal cannula therapy on mechanical ventilation duration in the pediatric intensive care unit
Source: PLoS One. 2024 Dec 13;19(12):e0315736. doi: 10.1371/journal.pone.0315736 (PMC12140079; doi:10.1371/journal.pone.0315736)

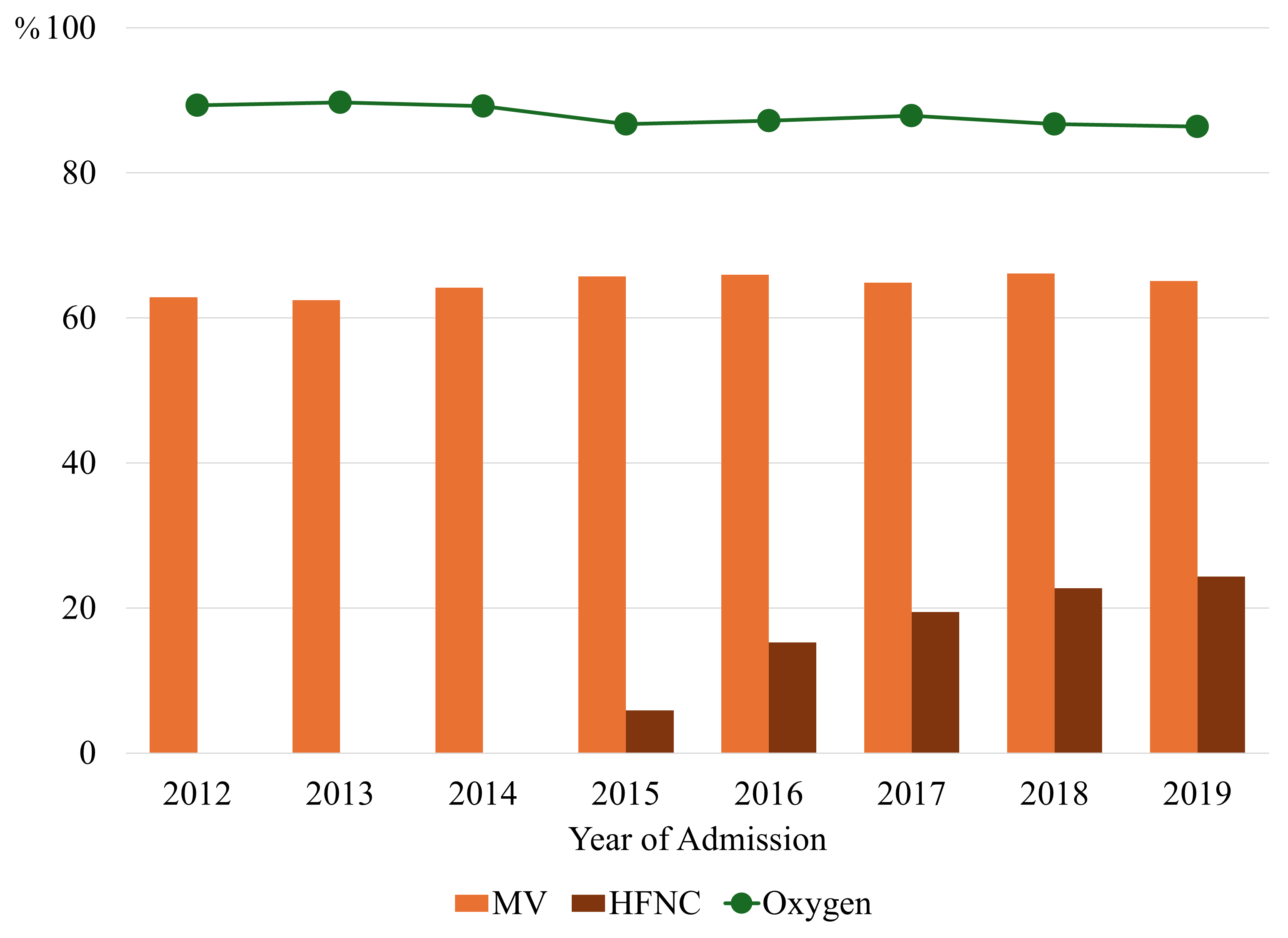

Supplement: S1 Fig — MV, mechanical ventilation; HFNC, high-flow nasal cannula therapy. (TIF) [file pone.0315736.s001.tif]

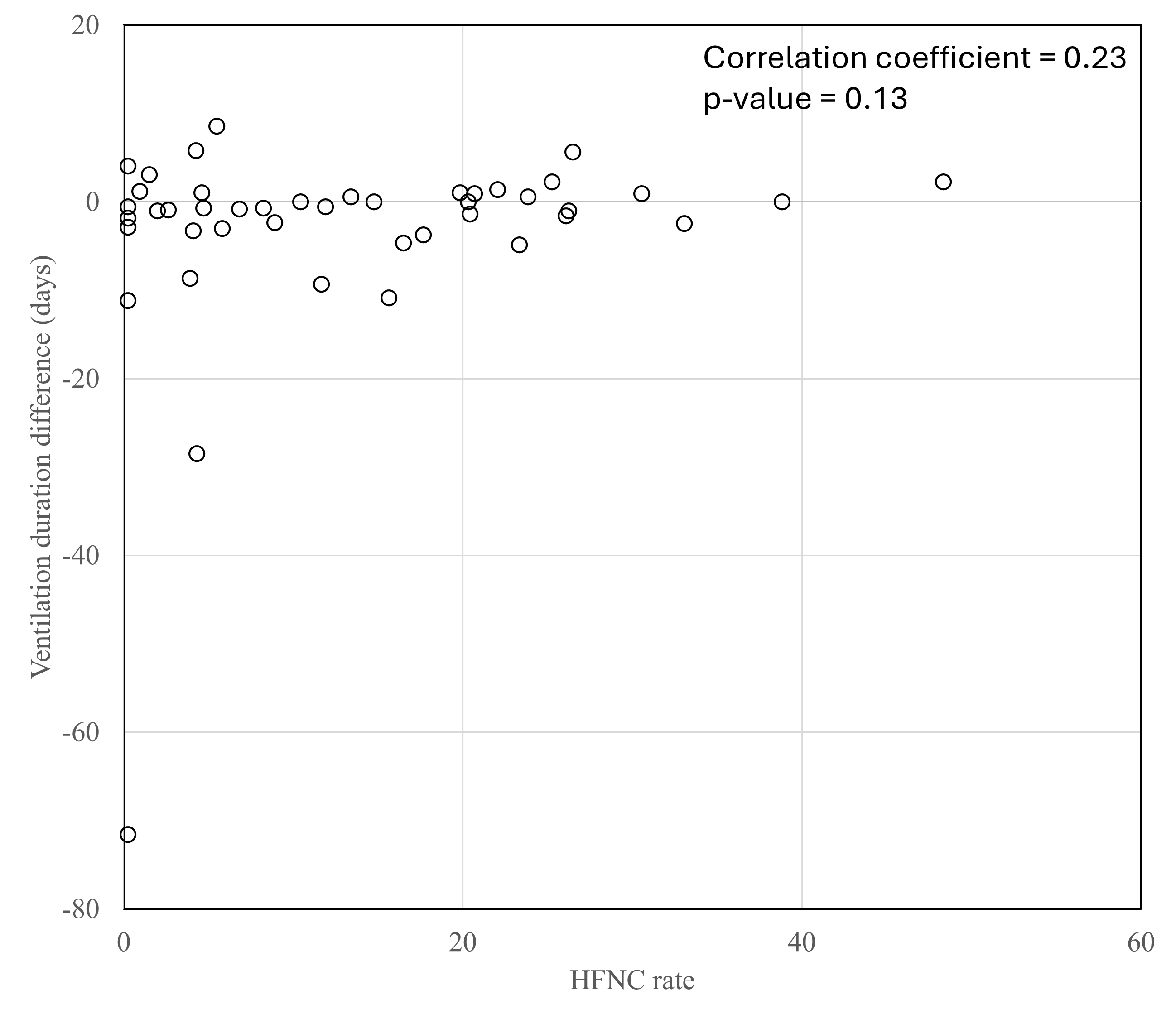

Supplement: S2 Fig — HFNC rate: rate of HFNC use among all respiratory support modalities (oxygen therapy, high flow nasal cannula, mechanical ventilation). Mechanical ventilation difference (days): mean ventilation duration of the post-HFNC period minus that of the pre-HFNC period in each hospital (negative value indicates a reduction in ventilation duration after HFNC application). HFNC, high-flow nasal cannula therapy. (TIF) [file pone.0315736.s002.tif]
