# Supplementary material for: Effect of high-flow nasal cannula therapy on mechanical ventilation duration in the pediatric intensive care unit
Source: PLoS One. 2024 Dec 13;19(12):e0315736. doi: 10.1371/journal.pone.0315736 (PMC12140079; doi:10.1371/journal.pone.0315736)
Supplement: S1 Table — (DOCX) [file pone.0315736.s003.docx]

**S1 Table. Number and percentage of patients with mechanical ventilation by subgroups**

|  | Pre-HFNC period (N=4,705) | Post-HFNC period (N=4,864) |
| --- | --- | --- |
|  | **Number (%)** | **Number (%)** |
| Overall | 3,047 (64.8) | 3,263 (67.1) |
| Age group |  |  |
| Infants (<1) | 1044 (68.3) | 1137 (68.4) |
| Children (1-11) | 1452 (66.5) | 1562 (69.0) |
| Adolescents (12-17) | 529 (53.2) | 532 (56.7) |
| Sex |  |  |
| Male | 1740 (64.9) | 1843 (66.5) |
| Female | 1321 (65.2) | 1407 (67.2) |
| Primary diagnosis |  |  |
| Congenital anomaly | 1410 (82.0) | 1577 (84.4) |
| Neoplasm | 377 (56.3) | 373 (57.0) |
| Neurologic disease | 275 (58.4) | 315 (60.8) |
| Respiratory | 274 (65.0) | 316 (65.9) |
| Circulatory disease | 304 (66.1) | 283 (74.4) |
| Injury | 137 (45.6) | 121 (42.4) |
| Gastrointestinal disease | 74 (59.8) | 95 (65.7) |
| Not elsewhere classified | 51 (42.1) | 67 (49.8) |
| Infectious disease | 34 (55.5) | 49 (58.5) |
| Others | 186 (52.0) | 156 (49.9) |
| Region |  |  |
| Seoul | 2202 (69.8) | 2228 (72.5) |
| Metropolitan | 440 (54.8) | 420 (54.7) |
| Rural | 432 (57.9) | 615 (60.2) |
| Admission department |  |  |
| Medical | 1267 (56.8) | 1409 (59.5) |
| Surgical | 1861 (75.3) | 1894 (75.9) |
| Surgery |  |  |
| No | 586 (50.0) | 631 (49.9) |
| Yes | 2508 (71.0) | 2654 (73.8) |
| Chest surgery | 782 (91.2) | 833 (86.4) |
| Intervention for critical care |  |  |
| Vasopressor | 2392 (80.2) | 2660 (80.2) |
| ECMO | 74 (91.8) | 63 (86.9) |
| Hemodialysis | 161 (80.8) | 165 (78.1) |

HFNC, high flow nasal cannula; ECMO, extracorporeal membrane oxygenation
