# Supplementary material for: Effect of high-flow nasal cannula therapy on mechanical ventilation duration in the pediatric intensive care unit
Source: PLoS One. 2024 Dec 13;19(12):e0315736. doi: 10.1371/journal.pone.0315736 (PMC12140079; doi:10.1371/journal.pone.0315736)
Supplement: S2 Table — (DOCX) [file pone.0315736.s004.docx]

**S2 Table. Mean and standard deviation of mechanical ventilation duration according to HFNC period by subgroup.**

|  | **Pre-HFNC period (N=4,705)** | **Post-HFNC period (N=4,864)** |
| --- | --- | --- |
|  | **Mean (SD)** | **Mean (SD)** |
| **Overall** | 5.6 (27.6) | 4.8 (14.9) |
| **MV status** |  |  |
| No MV used or MV ≤ 28 days | 2.8 (4.6) | 2.8 (4.6) |
| MV >28 days | 89.8 (126.6) | 64.4 (51.7) |
| MV used (> 0 day) | 8.6 (33.9) | 7.2 (17.8) |
| **Surgical status** |  |  |
| Overall surgical group | 5.9 (30.6) | 4.7 (14.1) |
| Chest surgery group | 3.1 (8.4) | 2.9 (7.9) |
| **Diagnostic subgroup** |  |  |
| Neurologic disease | 10.3 (60.6) | 6.3 (12.9) |
| Respiratory disease | 6.5 (23.9) | 7.9 (23.5) |
| Circulatory disease | 8.2 (36.3) | 5.5 (14.5) |

HFNC, high flow nasal cannula; MV, mechanical ventilation
