# Supplementary material for: Effect of high-flow nasal cannula therapy on mechanical ventilation duration in the pediatric intensive care unit
Source: PLoS One. 2024 Dec 13;19(12):e0315736. doi: 10.1371/journal.pone.0315736 (PMC12140079; doi:10.1371/journal.pone.0315736)
Supplement: S3 Table — (DOCX) [file pone.0315736.s005.docx]

**S3 Table. Number of patients who received mechanical ventilation who died within 28 days.**

|  | **Overall** | | **Pre HFNC period** | | **Post-HFNC period** | |
| --- | --- | --- | --- | --- | --- | --- |
|  | **No. overall** | **No. deaths** | **No. overall** | **No. deaths** | **No. overall** | **No. deaths** |
| **Overall** | 9242 | 416 (4.5) | 4541 | 203 (4.5) | 4701 | 213 (4.5) |
| **MV status** |  |  |  |  |  |  |
| MV used (> 0 day) | 5,983 | 384 (6.4) | 2883 | 188 (6.5) | 3100 | 196 (6.3) |
| Surgical status |  |  |  |  |  |  |
| Overall surgical group | 6,878 | 201(2.9) | 3399 | 99 (2.9) | 3479 | 102 (2.9) |
| Chest surgery group | 1921 | 10 (0.5) | 893 | 5 (0.6) | 1028 | 5 (0.5) |
| Diagnostic subgroup |  |  |  |  |  |  |
| Neurologic disease | 922 | 40 (4.3) | 437 | **15 (3.4)** | **485** | **25 (5.2)** |
| Respiratory disease | 859 | 20 (2.3) | 408 | 16 (3.9) | 451 | 4 (0.9) |
| Circulatory disease | 801 | 65 (8.1) | 437 | 31 (7.1) | 364 | 34 (9.3) |

HFNC, high flow nasal cannula; MV, mechanical ventilation
