# Supplementary material for: Effect of high-flow nasal cannula therapy on mechanical ventilation duration in the pediatric intensive care unit
Source: PLoS One. 2024 Dec 13;19(12):e0315736. doi: 10.1371/journal.pone.0315736 (PMC12140079; doi:10.1371/journal.pone.0315736)
Supplement: S4 Table — (DOCX) [file pone.0315736.s006.docx]

**S4 Table. Mean and standard deviation of ventilator-free days at day 28 according to HFNC period by subgroup.**

|  | **Pre-HFNC period (N=4,705)** | **Post-HFNC period (N=4,864)** |
| --- | --- | --- |
|  | **Mean (SD)** | **Mean (SD)** |
| **Overall** | 23.6 (7.8) | 23.5 (7.8) |
| MV status |  |  |
| MV used (> 0 day) | 21.3 (8.7) | 21.4 (8.6) |
| Surgical status |  |  |
| Overall surgical group | 23.9 (7.3) | 24.0 (7.2) |
| Chest surgery group | 25.2 (4.0) | 25.4 (3.8) |
| Diagnostic subgroup |  |  |
| Neurologic disease | 22.6 (8.9) | 22.0 (9.5) |
| Respiratory disease | 22.9 (7.8) | 22.3 (8.0) |
| Circulatory disease | 22.9 (9.2) | 22.0 (9.7) |

HFNC, high flow nasal cannula; MV, mechanical ventilation
