# Supplementary material for: Effect of high-flow nasal cannula therapy on mechanical ventilation duration in the pediatric intensive care unit
Source: PLoS One. 2024 Dec 13;19(12):e0315736. doi: 10.1371/journal.pone.0315736 (PMC12140079; doi:10.1371/journal.pone.0315736)
Supplement: Attachment — Submitted filename: Review of Manuscript PONE.docx [file pone.0315736.s007.docx]

Review of Manuscript PONE- D-24-30283

Authors, overall:

- Overall, excellent work and the recommendations ae minor and are simply to enhance your efforts.
- My recommendation is acceptance of your manuscript with consideration of the recommendations below to enhance the excellent quality of your work.
- A more detailed review of each of the above areas is outlined below.

Abstract

- Excellent overall description of background introduction, methods, results and conclusion.

Introduction

- Very good description of background issues and goals of study.
- Recommendations to Authors:
- Describe pediatric ICU as PICU as commonly seen in other studies to satisfy readers intuition.
- Describe other accepted means of HFNC use in pediatrics to include

acute hypoxemic respiratory failure, post-extubation, post-operative patients to include cardio-thoracic patients, obstructive lung disease such as asthma and bronchiolitis.

- Discuss prior work comparing efficacy and limitations of HFNC to standard oxygen therapy (SOT), nasal CPAP (n-CPAP) and NIPPV (Bi-PAP).
- Describe mechanical ventilation duration and ventilator-free days as the primary outcomes. Describe sub-group analysis of length of mechanical ventilation, surgical status and diagnostic subgroups as secondary outcomes.

Methods

- Excellent database source and description.
- Excellent inclusion criteria and exclusion of NICU patients in presence of known age and physiologic related differences in addition to age-related co-morbidities.
- Good description of VFD and “other variables”.
- Good description of intended primary outcomes.
- Appropriate statistical analysis.
- Recommendations to authors: none.

Results

- Good overall description would be enhanced by the following.
- Recommendations to authors:
- Figure 1 is not true flow diagram or reflective of patient selection and exclusion; rename as “annual description of respiratory support” and provide “p-values” to outline statistically significant difference between both eras (pre- and post- HFNC initiation).
- Table 1 – rename as baseline characteristics of study patients and categorize hospital, PICU length of stays and mortality as “Outcomes”.
- Paragraph 3 – list “Table 2” earlier…”In the adjusted model (Table 2) there was significant reduction” and not at the end of the paragraph so that readers can more easily refer to the table and note your accurate description of the result.
- Paragraph 4- list “Table 4” earlier…”When assessed by subgroup (Table 4), the adjusted duration…” and not at the end of the paragraph so that readers can more easily refer to the table and note your accurate description of the result.
- Page 10 - provide statistical data (p-values, correlation co-efficient) to support your likely correct contention that there was no association between rate of HFNC usage and change in mean mechanical ventilation duration in individual hospitals and briefly add the same information to Figure 2 to provide the same statistical absence of association.

Tables, Figures

- Page 26 – line 465 should read S2 Table, not S1 Table
- Page 26 – line 466 should read S1 Table, not S2 Table.

Discussion:

- ↑ use of HFNC since initiation 2015.
- Baseline characteristics - younger age, anomalies, rural location, vasopressor use.
- Key findings:

Increase use of mechanical ventilation.

Duration ↓if ≥ 28 days and in.

post-operative surgical and neurologic diagnosis.

No decrease in VFD in general but was present in surgical patients.

Implications:

- HFNC is effective in post-operative patients and those with prolonged mechanical ventilation (≥ 28 days).
- Facilitates availability of PICU beds in tertiary care centers.
- Facilitates transfer of those patients requiring higher level of care through avoidance of complications and risks of invasive mechanical ventilation during transport process.
- Absence of reduction in VFD is likely due to pragmatic use of HFNC in this study compared to prophylactic use in other or future studies.

Future work:

- Evaluate effectiveness of HFNC to increase VFD when used empirically to avoid mechanical ventilation and to earlier successful extubation.
- Multi-center, international study to evaluate generalizability regarding effective use of HFNC to facilitate reduction in duration of mechanical ventilation, availability of tertiary PICU beds through use at other urban community hospitals and rural locations.

Confidential Comments to Editor

Professor Grosek,

I have reviewed PLOS ONE manuscript PONE-D-24-30283.

I recommend that it be accepted for publication with minor revisions as presented to authors. Overall, the conception, design, objectives, methods, presented results, conclusions and implications are well presented and the revisions are minor to enhance the author’s excellent work.

I appreciate the opportunity to review this manuscript and remain available through my email address ([JLaham3@gmail.com](mailto:JLaham3@gmail.com)) for any further questions or issues.

Best regards

James Laham, D.O.
